# Supplementary material for: Potentially inappropriate prescriptions according to explicit and implicit criteria in patients with multimorbidity and polypharmacy. MULTIPAP: A cross-sectional study
Source: PLoS One. 2020 Aug 12;15(8):e0237186. doi: 10.1371/journal.pone.0237186 (PMC7423095; doi:10.1371/journal.pone.0237186)
Supplement: Attachment — Submitted filename: 2020-04-30 Response to reviewrs.docx [file pone.0237186.s006.docx]

April 30^th^, 2020

Dear Editor

Thank you very much for the detailed revision received.

We enclose our manuscript for your consideration after the changes you suggested.

We hope that the manuscript review meets the high standards of your Journal, and look forward to receiving your comments. If you have any questions, please do not hesitate contact us

We will address all reviewers’ comments below replying between the lines for each comment.

Best regards

María Eloísa Rogero-Blanco (corresponding Author)

**Response to reviewers**

Reviewer #1: Thank you for the opportunity to review this research work about the potentially inappropriate prescriptions in patients with multimorbidity and polypharmacy. I hope the following comments/ suggestions be useful in the development of this document.

**I want to suggest it is needed to review the entire document by a native translator. The document needs changes that increase clarity and facilitate its reading.**

Thank you very much for the suggestion regarding the language. We tried our best by translating and editing it by American Journal Expert. We attached the translation certification. We considered the American native speaker for the scope of this journal. We re-reviewed the translation and incorporated some changes to improve readability. Your suggestions have also been sent to AJE for clarification.

**Title: Second part of the title could be more precise. Please, consider: “Potentially Inappropriate prescriptions according to explicit and implicit criteria in patients with multimorbidity and polypharmacy. MULTIPAP: A cross-sectional study.”**

We have incorporated your proposed title

**Abstract: According to the “Submission Guidelines” the Abstract should not include citations and abbreviations, if possible. Please, check it.**

**- Line 44: Multi-PAP is written in capitals in the title. Please, you should unify it.** MULTIPAP has been corrected and unified.

**- Line 51: if you decide to include these cites, please, check how doing it.**

All abbreviations have been checked and revised to include. The abbreviation Potentially Inappropriate Prescription (PIP) has been removed from the abstract. The Medication Appropriateness Index (MAI) has been maintained as it is the most common name for the questionnaire, as well as the STOPP criteria.

**Introduction: The contents of the introduction need to be related and exposed following a somewhat brighter argument line.**

**- Line 66-67: please include the cite/s for this definition.** The reference has been included.

**- Lines 76, 97: the number must be before the "."** The number has been placed before the [ ]

**- Line 96: Change “In our country…” by “In Spain…”** The change has been made.

**- Line 97-98: Consider strengthening the rationale for this statement.** It has been deleted to simplify since it doesn’t follow the narrative structure of the paragraph.

**- Line 99: Rewrite in a proper way mean and standard deviation.** The change has been made.

**- Lines 99 to 106: Please, consider to examine this paragraph. It seems not clear enough. Something is missing in the last sentence.** The paragraph has been revised and the acronym PC has been replaced throughout the text by primary care to make it more reader-friendly.

**- Line 110, 112: Review the cite.** The cite has been revised. **- Lines 114-116: Please, consider to examine this paragraph. It seems not clear enough.** Paragraph has been restated to help understanding.  **- Line 124: Compare and concur the aim of the abstract and the introduction.** The aims have been compared and their wording unified.

**- Lines 125-126: Review the cites**. Lines 125 and 126 correspond to the wording of the objective, they do not contain cites.

**Material and methods:
- Line 148: the data were collected December 2016 – January 2017? Have authors thought about include data from 2019 or 2020?**

Sociodemographic and clinical data were obtained during the period of patient recruitment in the MULTIPAP study, which was conducted between December 2016 and January 2017 through an interview with each patient's family doctor. It is the baseline data of the study that are the object of interest of this work.

**- There were used the same method of data collection in patients and physicians? Please, clarify the method and variables for each group of participants.**

The method of data collection on patients and physicians was not the same.

The method of collecting data on patients and doctors was not the same. Data on professionals (age, sex, length of physician career, and being postgraduate medical supervisor) were recorded by each physician in the data collection notebook after signing their commitment to collaborate in the study. Subsequently, sociodemographic and clinical data on patients were collected through an interview with each patient's general practitioner after signing the informed consent.

**Results
- What is the range age of the patients?**

Since inclusion criteria were strict trying to look for “young seniors”, the age range is exactly the same as the inclusion criteria, being 65-74 years old. For this reason we didn’t include in the manuscript. Interquartile range was 68-72.

**- Line 236: It is explained that “digestive system/metabolism” is one of the most popular group prescriptions, but in table 2, this group is identified as “metabolism and alimentary tract”. Please, check and change this issue**.

The change has been made

**Discussion**

-**Line 349: The authors affirm the absence of studies with MAI quantitative data based on a 2013 article. Please check that the absence of evidence exists currently.**

We’ve reviewed the text and changed it to reflect what happens in the Spanish population. We included a more updated reference regarding the most alike population (French study by Gilbert). Citations has been included and updated.

**The conclusion section is missing.**

Following the sections proposed by the journal Plos one, Conclusions (optional) the section has not been included.

Following the reviewer's proposal, we have incorporated an epigraph “Implications of the study findings” synthesizing the conclusions. If you consider that a specific section of conclusions is more appropriate, we will proceed to do so.

**Tables: check them and give the format according to the “Submission Guidelines”.** Reviewed

**Table 1: Complete with additional results (age range for patients & physicians, male gender, …)** Included in the tables

**Table 3: Consider to remove column 1.**

We have removed column 1.  **References: Check and make changes following the “Submission Guidelines”.** Cheked

**Reviewer #2: General comments**

**Pay attention to punctuation for instance in lines 76, 97.**The number has been placed before the [ ]

**Introduction
- Lines 67-68, can the authors point to a reference to back up it?**. It has been pointed out since it’s a shared comment with reviewer 1

**Method**

**- Why did you choose this inclusion criterion for the age of 74, for your study?**

In Spain, in the context of the Strategy of the Ministry of Health, Social Policy and Equality to improve the care of polymedicated chronic patients, programs aimed at polymedicated elderly patients (>75 years) have been implemented both nationally and in various regions. However, patients over 75 years old are only a part of the population with multi-morbidity. Patients who are sociologically define as young seniors (65-74 years old) have a high prevalence of multimorbility and polymedication and, although evidence on the effectiveness of interventions on this population group is even more limited, this is an age group with an important potential for early intervention. For this reason our study group focuses on this age range.

**- Delete line 141, “Participating physicians did not receive financial compensation”.** The line has been deleted

**- Line 143: “from three Spanish regions …”, how were these regions selected? Based on which method?**

The three regions are Andalusia, Aragon and Madrid. They represent three regions geographically located in the south, centre and north of the country respectively. In each one, the Cooperative Research Network on Services and Chronic Diseases (REDISSEC) of the Carlos III Health Institute [www.redisecc.com](http://www.redisecc.com) has a research group with proven research experience on patients with multimorbility and chronic diseases. Our research approach both, the epidemiological perspective of the characterization of chronicity and the evaluation of the effectiveness of interventions in patients with chronic diseases in primary care.

These groups from the three regions obtained funding in competitive public calls for the MULTIPAP study, which includes cross-sectional studies such as the one proposed here, clinical trials, and cohort studies. [www.multipap.es](http://www.multipap.es)

**- Line 155: First time an abbreviation is used, the term should be spelled out in full.** The term has been included in its complete form. The Organisation for Economic Co-operation and Development (OECD)

**Results
- This section needs minor rectifications. Please consult with a statistician for how the section should be journalistic way written (lines 223-131).**

Several members of the research team are statisticians. We understand that you are referring to the wording of the paragraph in the lines **(lines 223-231).** Following your suggestions we have simplified the wording of the paragraph.

**Discussion
- Line 343: delete (5 studies).** The words have been deleted

**- Lines 351 (… differ from those found in the literature.), 355 (… than in the previous studies): can the authors point to some reference to back up them?**

The three citations supporting those statements have been relocated according to the specific section.

**Limitations
- Line 427, delete (Among the main limitations) and then add all limitations of your study.**

“Among the main limitations” has been deleted

**Also, please add “Conclusion” section and mention main findings of your study, implications.**

Following the sections proposed by the journal Plos one, Conclusions (optional) the section has not been included.

Following the reviewer's proposal, we have incorporated an epigraph “Implications of the study findings” synthesizing the conclusions. If you consider that a specific section of conclusions is more appropriate, we will proceed to do so.
