# Supplementary material for: Potentially inappropriate prescriptions according to explicit and implicit criteria in patients with multimorbidity and polypharmacy. MULTIPAP: A cross-sectional study
Source: PLoS One. 2020 Aug 12;15(8):e0237186. doi: 10.1371/journal.pone.0237186 (PMC7423095; doi:10.1371/journal.pone.0237186)
Supplement: Attachment — Submitted filename: Response to reviewrs_19_06_2020.docx [file pone.0237186.s007.docx]

Jun 19^th^, 2020

Dear Editor

Thank you very much for the new revision received.

We are enclosing our manuscript for your consideration after the changes you suggested.

We hope that the review of the manuscripts will meet your suggestions and we look forward to receiving your comments. If you have any questions, please do not hesitate to contact us

We addressed all reviewers' comments below.

Best regards.

María Eloísa Rogero-Blanco (Corresponding author)

**Response to reviewers**

Reviewer #1: Dear author thanks for increasing manuscript quality with your efforts. Nevertheless, some of the changes suggested are missing yet.

**Line 39-41**: **sample size has been removed from the abstract. Please, add it again.**

Sample size has been included

**Line 51-52: Add the bibliographic reference of each criterion: STOPP 2014, STOPP 2008, Beers 2019 & Beers 2015.**

After reviewing journal style, Abstract section, along with other sections, does not accept bibliographic references. We understood, and after an editor consultation, that what was asked was to include acronyms definition for the abbreviations included in the abstract.

**Introduction: I suggested in previous inform: "The contents of the introduction need to be related and exposed following a somewhat brighter argument line" and nothing has been done with this issue.**

The argument line in the introduction has been changed

**In response to reviewers, it is answered to my question about the method of data collection in patients and physicians. Please, include in the manuscript, in the same manner, the explanation given to reviewers.**

The explanation given to the reviewers previously has been included in methods section.

**Also, I would like to request again that be included the range age of patients in the results section, despite you explained it was an inclusion criteria.**

The age range was already included in Table 1 and has been moved to results section as suggested.

**Reviewer 2 and 1 agree with a conclusion section is needed in the manuscript. Please, consider to include it.**

We have included the conclusions section

Reviewer #2: The only challenge to the paper is that it needs a bit of editing for use of English grammar. With that editing I recommend publication.

**Table 1: Substitute “Female, Male” instead of “Women, Men”.**

The change has been made

**Lines 408-410: Grammar needs to be reviewed.**

We have reviewed the grammar in these lines
