# Supplementary material for: Potentially inappropriate prescriptions according to explicit and implicit criteria in patients with multimorbidity and polypharmacy. MULTIPAP: A cross-sectional study
Source: PLoS One. 2020 Aug 12;15(8):e0237186. doi: 10.1371/journal.pone.0237186 (PMC7423095; doi:10.1371/journal.pone.0237186)
Supplement: Attachment — Submitted filename: Response to reviewrs_16_07_2020.docx [file pone.0237186.s008.docx]

Jul 17^th^, 2020

Dear Editor

Thank you very much for the new revision received.

We are enclosing our manuscript for your consideration after the changes you suggested.

We hope that the review of the manuscripts will meet your suggestions and we look forward to receiving your comments. If you have any questions, please do not hesitate to contact us

We addressed all reviewers' comments below.

Best regards.

María Eloísa Rogero-Blanco (Corresponding author)

**Response to reviewers**

Reviewer #1:

**Lines 40-42- In order to increase clarity, this is my suggestion: "[...] according to American Geriatrics Society Beers Criteria® (2015, 2019), the Screening Tool of Older Person’s Prescription -STOPP- criteria (2008, 2014), and the Medication Appropriateness Index -MAI- criteria**

The change has been made. It is true that it is clearer
